# The effects of microgravity on differentiation and cell growth in stem cells and cancer stem cells

**DOI:** 10.1002/sctm.20-0084

**Published:** 2020-04-30

**Authors:** Daniela Grimm, Markus Wehland, Thomas J. Corydon, Peter Richter, Binod Prasad, Johann Bauer, Marcel Egli, Sascha Kopp, Michael Lebert, Marcus Krüger

**Affiliations:** ^1^ Department of Microgravity and Translational Regenerative Medicine Otto von Guericke University Magdeburg Germany; ^2^ Clinic for Plastic, Aesthetic and Hand Surgery Otto von Guericke University Magdeburg Germany; ^3^ Department of Biomedicine Aarhus University Aarhus Denmark; ^4^ Department of Ophthalmology Aarhus University Hospital Aarhus Denmark; ^5^ Department of Biology Friedrich‐Alexander‐University Erlangen‐Nuremberg Erlangen Germany; ^6^ Max Planck Institute of Biochemistry Planegg‐Martinsried Germany; ^7^ Institute of Medical Engineering, Space Biology Group Lucerne University of Applied Sciences and Arts Hergiswil Switzerland; ^8^ Space Biology Unlimited SAS Bordeaux France

**Keywords:** cancer stem cells, microgravity, multicellular spheroids, organoids, random positioning machine, rotating wall vessel, spaceflight, stem cells, tissue engineering

## Abstract

A spaceflight has enormous influence on the health of space voyagers due to the combined effects of microgravity and cosmic radiation. Known effects of microgravity (μ*g*) on cells are changes in differentiation and growth. Considering the commercialization of spaceflight, future space exploration, and long‐term manned flights, research focusing on differentiation and growth of stem cells and cancer cells exposed to real (r‐) and simulated (s‐) μ*g* is of high interest for regenerative medicine and cancer research. In this review, we focus on platforms to study r‐ and s‐μ*g* as well as the impact of μ*g* on cancer stem cells in the field of gastrointestinal cancer, lung cancer, and osteosarcoma. Moreover, we review the current knowledge of different types of stem cells exposed to μ*g* conditions with regard to differentiation and engineering of cartilage, bone, vasculature, heart, skin, and liver constructs.

## Graphical abstract

Stem cells exposed to real or simulated microgravity show changes in growth and differentiation. The application of microgravity in cell biology represents a new technology used in the field of cancer research and translational regenerative medicine. It extends the current knowledge in the engineering of organoids, spheroids, or tissues (bone, liver, and heart among others) with and without scaffolds.

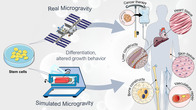

**Significance statement**

Microgravity provides a unique environment for cell culture and has been shown to induce cellular changes and processes that could not be achieved under normal gravitational conditions. Over the past years, it has therefore gained increasing importance in different research fields such as cancer research, where microgravity may help understanding and suppressing tumor metastasis, or tissue engineering, where it induces the scaffold‐free formation of three‐dimensional multicellular spheroids. This review will give a concise overview of the current knowledge on the effects of microgravity on stem cells and cancer stem cells, and will highlight novel therapeutic options derived from it.

## 1 INTRODUCTION

Microgravity (μ*g*) induces a large number of changes in specialized cells and stem cells.^1^, ^2^, ^3^ Experiments in orbit on the International Space Station (ISS), on unmanned spacecrafts or sounding rockets and on Earth using devices simulating μ*g* such as the random positioning machine (RPM), or a clinostat (CN) demonstrated among others alterations of the cytoskeleton,^4^, ^5^, ^6^, ^7^ changes in the composition of the extracellular matrix,^8^ focal adherence complex,^6^ proliferation,^9^ differentiation,^10^ and growth behavior of the cells.^1^ These changes were observed in different cell types.^1^

Gravitational biology and space medicine are currently of high interest and a hot topic in space research. A PubMed search on 27 February 2020 gave 9010 matches for the term “weightlessness,” 11 338 matches for “microgravity,” 549 matches for “microgravity and differentiation,” and 273 matches for “microgravity and stem cells.”

**FIGURE 1:**
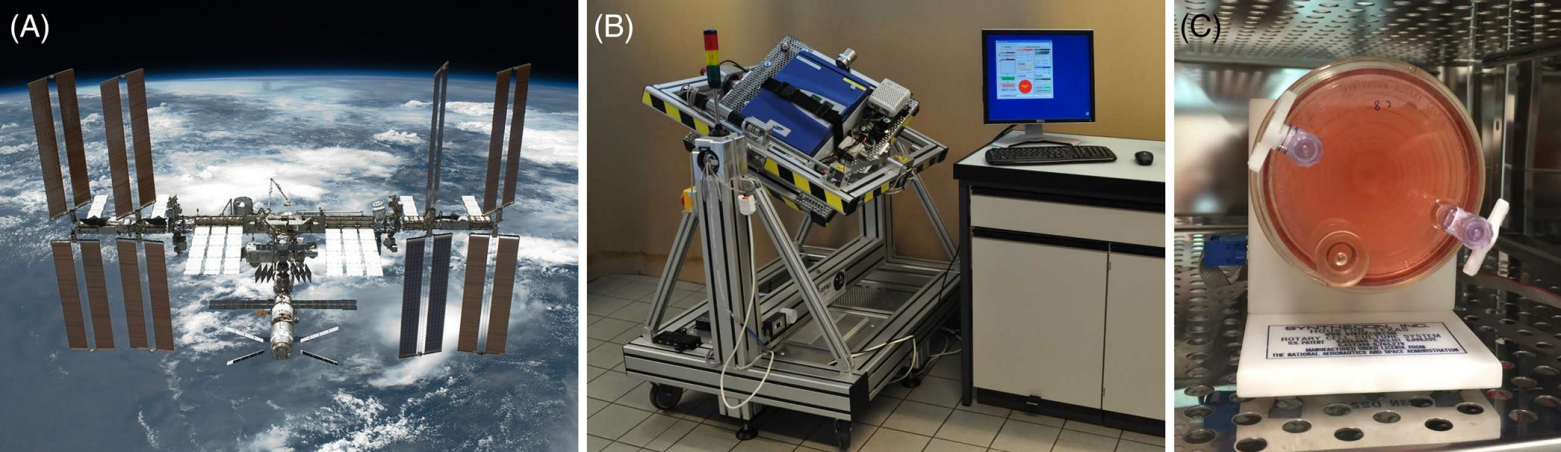
Platforms and devices used for stem cell research under real and simulated microgravity conditions. A, The International Space Station enables long‐term cell culture in real microgravity. B, Custom‐made random positioning machine (RPM) for the cultivation of mammalian cells under simulated microgravity conditions.^20^ C, The rotating wall vessel (RWV) is another type of microgravity simulator suitable for suspension cell cultures or adherent cells on microcarrier beads

An important aspect is the differentiation and redifferentiation potential of benign and malignant cells grown under simulated and real μ*g* conditions. Endothelial progenitor cells exposed to μ*g* revealed an improved angiogenic potential.^11^ Human‐induced pluripotent stem cell‐derived cardiomyocytes (hiPSC‐CMs) cultured aboard the ISS exhibited alterations in calcium handling and showed 2635 differentially expressed genes among flight, postflight, and ground control samples.^12^ In particular, genes involved in mitochondrial metabolism were differentially regulated.^12^ Recently, human blood‐derived stem cells had been investigated on the ISS. Compared with cells cultivated on Earth, a reduced expression of Sox2, Oct3/4, Nanog, and E‐cadherin was measured in space, together with an earlier osteogenic differentiation.^13^ In addition, cancer cells have proven to re‐differentiate after exposure to r‐ and s‐μ*g*.^10^, ^14^, ^15^ Studies in μ*g* demonstrated that cells exposed to gravitational unloading changed their growth behavior and started growing in a two‐ (2D) and three‐dimensional (3D) manner in space and on the rotating wall vessel (RWV), the RPM, CN, or on rotary cell culture systems (RCCS) (Figure 1).^1^, ^16^, ^17^, ^18^, ^19^, ^20^

**TABLE 1:** Summary of selected articles addressing research on cancer stem cells and stem in vitro cultured under simulated microgravity, ordered by organ/tissue

Cell line	Organ/tissue/cell type	Space or μ*g*‐simulating device	Findings	References
H460	Non‐small cell lung cancer	RPM	Increase in apoptosis, CSCs lost their stemness features, downregulated *Nanog* and *Oct4* genes	Pisanu et al^15^
HCT116	Colorectal cancer	RCCS	CSC; CD133/CD44 dual positive cells, giant cancer cells housing complete nuclear localization of YAP	Arun et al^14^, ^48^
SAOS‐2, HOS, U2OS T98G, U87MG Du145, LNCap H23 Hep3b Hela Mewo, HO‐1	Osteosarcoma Glioblastoma Prostate cancer Lung cancer Hepatocarcinoma Cervical carcinoma Melanoma	HFB	CD133^+^ cells from cancer cell lines	Kelly et al^55^
Rabbit MSCs	Cartilage tissue constructs	RWV	Cartilage nature confirmed by aggrecan and collagen types I and II gene expression as well as by toluidine blue and safranin‐O staining	Ohyabu et al^57^
Rabbit adipose‐derived stem cells and bone marrow stromal cells	Cartilage	RCCS, novel cell carrier derived from natural cartilage ECM	Improved the induction of stem cell chondro‐genesis as well as in vivo repair of cartilage lesions in a rabbit model	Yin H et al^62^, ^63^
hMSCs	Bone marrow, osteogenic lineage	RWV	Not suitable for a potential application in cartilage repair	Mayer‐Wagner et al^66^
hBMSCs	Bone marrow	ISS	r‐μ*g* stresses reverting to a quiescent state	Bradamante et al^77^
hADMSCs	Adipose tissue	RPM	Oxygen is a key player for cytoskeletal alterations and modulation of gene expression	Versari et al^118^
Rat BMSCs	Bone‐like tissue	STLV bioreactor, chitosan/hydroxyapatite, 28 days	BM‐MSCs‐C/HAp composite microbeads	Koç Demir et al^73^
BMSCs	Bone marrow	Clinostat	Depolymerized actin cytoskeleton inhibits osteogenic differentiation of BMSCs through impeding nuclear aggregation of TAZ	Chen et al^76^
CD34‐positive human cord blood stem cells (CBSC)	Vascular tubular assemblies	RWV, with or without Cytodex‐3 microcarrier beads and VEGF	Transdifferentiation into the vascular endothelial cell phenotype and assembling into 3D tissue structures	Chiu et al^80^
BMSCs	Endothelium‐like cells	Clinostat	Endothelial‐specific molecules (Flk‐1 and vWF) positive	Zhang et al^82^
EPCs	PBMNC	3D clinostat	Most significant increase in CD34+ and double positive Dil‐Ac‐LDL‐FITC‐Ulex‐Lectin cells, both EPC markers. Enhancing the number and angiogenic potential of EPCs	Hagiwara et al^9^
Pluripotent stem cell‐derived cardiomyocytes	Heart	ISS	Alterations in hiPSC‐CM calcium handling showed 2635 differentially expressed genes	Wnorowski et al^12^
CPCs	Cardiac tissue	ISS, 2D Clinostat	Hippo signaling; upregulation of downstream genes: *YAP1* and *SOD2*	Camberos et al^85^
Adult and neonatal CPCs	Cardiac repair	ISS	Only neonatal CPCs showed an increased expression of early developmental markers and an enhanced proliferative potential	Baio et al^84^
IMR90 iPSCs, hESCs (H7 and H9)	Progenitor cardiac spheres	RPM	Progenitor cardiac spheres (RPM) result in efficient generation of highly enriched hPSC‐CMs. Increase in proliferation and viability of CPCs	Jha et al^89^
Mouse ESCs	Mouse embryo	2D Clinostat	Deregulation of genes involved in heart development and inhibition of cardiomyocyte specific genes	Shinde et al^46^
PICM‐19	Pig liver tissue	Spaceflight, STS‐126 mission	In vitro model for assessing liver function in μ*g*. Minor differences between 1*g* and μ*g*	Talbot et al^109^
ADSCs	Subcutaneous adipose tissue	μ*g* bioreactor	Stemness properties, including self‐renewal and multipotency differentiation capacities, were enhanced by spheroid forma‐tion in μ*g*. Spheroid‐derived ADSCs showed more effective potentials to rescue liver failure	Zhang et al^108^
HepG2 Human biliary tree stem/progenitor cells (hBTSCs)	Hepatocyte carcinoma	RCCS	s‐μ*g* promotes the formation of 3D cultures and stimulates pluripotency and glycolytic metabolism in human hepatic and biliary tree stem/progenitor cells	Costantini et al^105^
Human epidermal stem cells (hEpSCs)	Epidermis‐like structure	RWV bioreactor, Cytodex‐3 microcarriers	hEpSCs aggregated on the microcarriers and formed multilayer 3D epidermis structures	Lei et al^94^
ADSCs	Adipose tissue	2D clinostat, CTGF	Differentiation to fibroblast cells. *Col1* and *ColIII*, *MMP1*, *ITGB1*, and *FSP1* gene expression changes involved	Ebnerasuly et al^95^

Abbreviations: 2D, two‐dimensional; 3D, three‐dimensional; AD(M)SCs, adipose tissue‐derived (mesenchymal) stem cells; BMSCs, bone marrow mesenchymal stem cells; *Col1*, collagen type I gene; *ColIII*, collagen type III gene; CPCs, cardiac progenitor cells; CTGF, connective tissue growth factor; ESCs, embryonic stem cells; EPCs, endothelial progenitor cells; FLK1, fetal liver kinase 1; *FSP1*, fibroblast‐specific protein 1 gene; hBTSCs, human biliary tree stem/progenitor cells; HFB, hydrodynamic focusing bioreactor; MCS, multicellular spheroids; *MMP1*, matrix metalloproteinase 1 gene; PBMNCs, peripheral blood mononuclear cells; RCCS, rotary cell culture system; RPM, random positioning machine; RWV, rotating wall vessel; r‐μ*g*, real microgravity; s‐μ*g*, simulated microgravity; SOD2, superoxide dismutase 2; STLV, slow turning lateral vessel‐type rotating bioreactor; TAZ, PDZ‐binding motif; VEGF, vascular endothelial growth factor; vWF, von Willebrand factor; *YAP1*, yes‐associated protein 1 gene.

Thus, it is possible to engineer scaffold‐free and scaffold‐containing 3D tissues such as preliminary vessels, cartilage, liver and bone pieces, and other organoids from different cell types using μ*g*.^1^ Currently, a large number of publications reported about the behavior of cancer cells in μ*g*, but little information exists about the behavior of cancer stem cells (CSCs) in μ*g*. Recently, investigations on colorectal CSCs were possible when HCT116 cells were exposed to a RCCS. Microgravity conditions provide a new method to study the biology of cancer stemness.^14^

This concise review will focus on the current knowledge of the impact of μ*g* on differentiation and cell growth in stem cells and CSCs.

## 2 DEFINITION OF CSCs

CSCs represent a subset of cells within the heterogeneous bulk of liquid and solid tumors.^21^ The term CSC was introduced for the first time by Dr John E. Dick and colleagues more than 25 years ago in their revolutionary study of certain kinds of human leukemia.^22^ CSCs share features with somatic stem cells, and therefore they possess characteristics of asymmetric division, self‐renewal, quiescence, and differentiation.^23^ In addition, these cells own the ability to replicate the parental tumor upon transplantation into a host as shown by Dick et al.^22^ CSCs also play a pivotal role in tumor pharmacological resistance due to upregulation of drug efflux transporters and DNA repair components.^21^, ^23^ Besides leukemia, CSCs have been identified in numerous tumor types, including those of the brain, breast, prostate, kidney, lung, and colon.^23^, ^24^ Accumulating evidence suggests mutations and epigenetic alterations in some tumor cells as main drivers leading to the establishment of CSCs.^25^ Microenvironment‐driven selection has also been implicated in the emergency of resistant cells with stem‐like characteristics.^26^ As CSCs are highly resistant to current therapeutic approaches, and therefore the main reason for cancer relapse, these cells are considered the prime therapeutic target for cancer treatment.^25^, ^27^, ^28^, ^29^

## 3 CSCs EXPOSED TO MICROGRAVITY

Little is known about the behavior of CSCs exposed to μ*g* conditions, but cancer researchers have started to work on this important topic.

Tumor cell heterogeneity and the presence of CSCs are responsible for a poor outcome of cancer patients and that metastatic breast cancer remains currently incurable, despite the progress in their detection and treatment.^30^ Breast CSCs have been identified as CD44^high^, CD24^low^, or aldehyde dehydrogenase positive (ALDH^+^).^31^, ^32^ Breast, thyroid, and prostate cancer cells differentiate and show various morphological alterations, change their growth behavior when grown in space or under s‐μ*g*.^33^, ^34^, ^35^, ^36^ The cancer cells differentiate into two phenotypes in the μ*g* environment. One part grows adherently on the bottom of the cell culture flasks and the other one assembled to 3D spheroids.^33^, ^34^, ^36^, ^37^ Multicellular spheroids (MCS) represent an intermediate metastasis model in its complexity between a monolayer culture and the in vivo tumor. These 3D MCS have the features of the primary tumor and also exhibit stem‐like features.^38^ Therefore, μ*g*‐engineered spheroids of different cancer types may represent an excellent model for detailed CSCs research.

Focused research on CSCs exposed to μg is published for various types of tumors.

### 3.1 Lung cancer

As in most other tumors, lung CSCs exhibit an increased expression of cell surface markers CD44 and CD133. Alternatively, lung CSCs may be purified using functional assays like ALDH^+^ ^8^ and side population (SP) assays.^39^ Several studies have investigated the influence of reduced gravity on lung cancer cell behavior.^40^, ^41^, ^42^ However, only few studies, to our knowledge, have reported an impact of s‐μ*g* on lung CSCs. One prominent example is the study by Pisanu et al in which lung CSCs, enriched from the stable non‐small cell lung cancer cell line H460, were subjected to s‐μ*g* obtained by means of an RPM.^15^ The study provides evidence that lung CSCs are committed to selective differentiation when subjected to reduced gravity. Furthermore, the study also reported increased apoptosis of CSCs incubated on the RPM compared to 1*g* controls. Collectively, these data suggest that lung CSCs, similar to somatic stem cells, are rescued from their quiescent state and lose their stemness default state when cultured in μ*g*. Consistent with this observation, a decrease in ALDH levels as well as downregulation of *Nanog* and *Oct4* genes were observed. Intriguingly, the μ*g*‐induced traits were stably attained and conserved by CSCs when cells were returned to 1*g* conditions.^15^

### 3.2 Gastrointestinal tumors

The gastrointestinal tract comprises stomach, colon, small intestine, rectum, and pancreas. During embryogenesis, these organs develop from stem cells and even in adults relevant stem or progenitor cells are persisting.^43^ They are capable to keep up the integrity of these organs, but may also be the origin of cancer development.^44^ Therefore, stem cell behavior is intensively investigated by a number of methods including exposure to s‐ or r‐μ*g*.^45^

However, little is known about the μ*g*‐dependent behavior of stem cells of the gastrointestinal tract. Whether the downregulation of the tropomyosin 1 gene (*Tpm1*) in stem cells^46^ is responsible for downregulated *Tpm1* gene in gastrocnemius muscle of mice observed after a 12‐day lasting spaceflight remains to be proven.^47^ Until now, Arun et al published a report about CSCs included in a population of colorectal cancer HTC116 cells.^48^ They cultured the whole population on a RCCS and observed that the percentage of CSCs expressing CD133 and CD44 simultaneously increased within the cell population. Their work suggests that μ*g* affects the growth/differentiation control elements FOXO3/PTEN/AKT.^14^ In addition, Devarasethy et al used an RWV bioreactor to form tumor organoids consisting of human hepatocytes, mesenchymal stem cells (MSCs) and colon carcinoma HCT116 cells. The subsequent investigation of the organoids revealed that the ratio of stem cells influenced the growth rate of the colon carcinoma cells.^49^

Stem cells are also present within adult human and mice pancreas cell populations.^50^, ^51^ If pancreatic cells are singularized and cultured within a hanging drop culture, they form pseudoislets with improved physiological properties.^52^ The result could be explained by the hypothesis that 3D spheroid formation favors the growth of progenitors, as it has been shown by Samuelson and Gerber,^53^ who successfully induced differentiation of mouse pancreatic progenitor cells by culturing them in a RWV bioreactor. Furthermore, if human pancreatic islets were predispersed and cocultured with stem cell such as amniotic epithelial cells (AECs) on a rotational cell culture system, islet:AEC aggregates were formed with improved insulin secretory capacity.^54^

### 3.3 Osteosarcoma and other tumors

Kelly et al investigated human osteosarcoma cells (SAOS‐2 cells) exposed to the NASA‐developed hydrofocusing bioreactor (HFB) and to the RCCS.^55^ The authors showed that CSCs were stimulated to proliferate when they are cultured in μ*g* conditions produced by the HFB. In addition, μ*g* sensitized CSCs to chemotherapeutic agents.^55^ Moreover, the authors investigated various cell types on the HFB such as osteosarcoma cells (SAOS‐2), prostate cancer cells, lung cancer cells, and melanoma cells and demonstrated that HFB exposure increased CD133‐positive cell growth from various cell lines compared with the RCCS vessel and to normal gravity control.^55^

## 4 MICROGRAVITY INFLUENCES DIFFERENTIATION AND GROWTH BEHAVIOR OF STEM CELLS

### 4.1 Use of stem cells for cartilage tissue engineering

Considering the overall development of progressively aging societies, the impact of cartilage damage and accompanying disorders such as osteoarthritis, rheumatoid arthritis, or intervertebral disk damage is steadily growing. One main problem of the treatment of cartilage lesions is the poor regenerative capacity of this tissue due to its avascularity and relatively low cell density, which is further augmented by the fact that the surviving chondrocytes shift their metabolic activity toward predominantly degenerative processes.^56^ For applications in regenerative medicine, it is therefore of high interest to circumvent these problems and to stimulate cartilage repair. One approach for this is the use of stem cells (Table 1).

MSCs derived either from bone marrow or from adipose tissue are most widely used. In general, s‐μ*g* seemed to be beneficial in promoting the differentiation of MSC into a chondrogenic phenotype. Ohyabu et al observed large tissue constructs after a 4‐week culture of rabbit MSCs in an RWV bioreactor. Their cartilaginous nature was confirmed by qPCR analyses of aggrecan and collagen types I and II gene expression as well as by toluidine blue and safranin‐O staining.^57^ Other authors described similar findings, where cultivation of MSCs under s‐μ*g* conditions led to a higher expression of chondrogenic markers than in static control groups and to a better quality cartilage tissue compared with standard 3D culture techniques.^58^, ^59^, ^60^ It has been suggested that these effects are mediated via the p38 MAPK pathway.^61^ Furthermore, simulated μ*g* was also reported to potentiate the proliferative capacity of MSCs.^60^

Yin et al have introduced the use of decellularized cartilage extracellular matrix‐derived particles as scaffolds for the culture of rabbit adipose‐derived stem cells as well as bone marrow stromal cells and found that s‐μ*g* conditions greatly improved the induction of stem cell chondrogenesis as well as in vivo repair of cartilage lesions in an animal model.^62^, ^63^ Another coculture approach used primary meniscus cells and MSCs. s‐μ*g* strongly increased cartilage matrix formation, the expression of hypertrophic differentiation markers COL10A1 and MMP‐13, and suppressed the hypertrophic differentiation inhibitor, gremlin 1.^64^ Interestingly, a recent study with Indian hedgehog and Sonic hedgehog transfected MSCs revealed that s‐μ*g* significantly promoted the differentiation of MSCs into chondrocytes and also inhibited chondrocyte hypertrophy and aging during chondrogenesis.^65^

It should be noted, however, that not all authors have found a promoting effect of s‐μ*g* on the chondrogenic differentiation of MSCs. In their experiments exposing human MSCs to s‐μ*g*, Mayer‐Wagner et al observed a lowered hypertrophy, but also a reduced chondrogenic potential, which could be in part counteracted by a low‐frequency electromagnetic field,^66^, ^67^ emphasizing the necessity for further research in this promising field.

### 4.2 Stem cells for the engineering of bone‐like tissues

It is well known that μ*g* influences calcium, sodium, and bone metabolism of humans in space. Bone loss and spaceflight‐induced osteopenia are problematic issues for long‐term space missions and future space exploration.^68^ Bone loss and radiation are the most substantial health risks of astronauts and should be managed by efficient countermeasures.^68^

**FIGURE 2:**
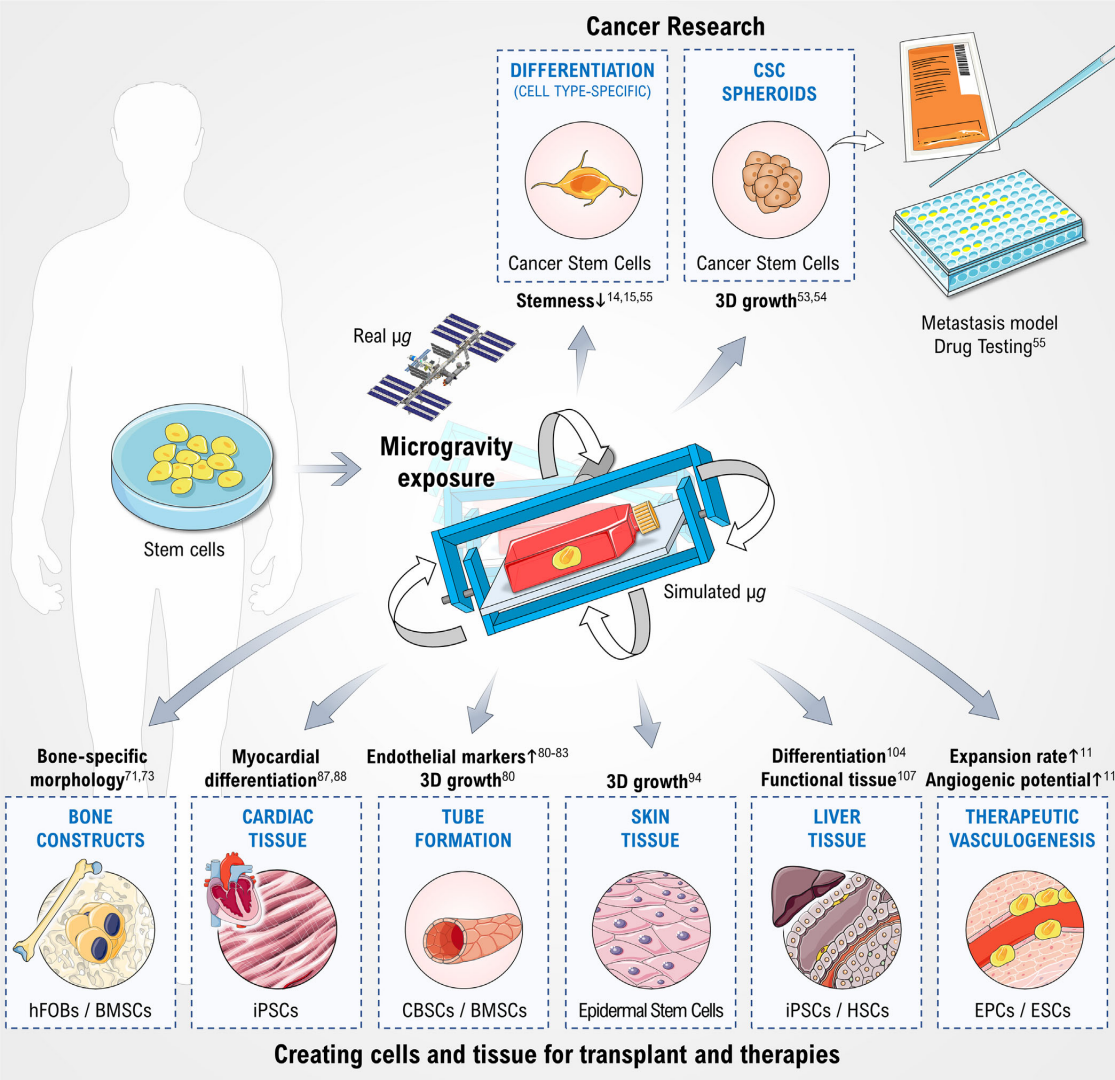
Effects of real or simulated microgravity on stem cell cultures (lower panel) and cancer stem cells (upper panel) together with the possible applications in medicine. Arrows indicate increases/reductions. BMSC, bone marrow‐derived stem cell; CBSC, umbilical cord blood stem cell; CSC, cancer stem cell; EPC, endothelial progenitor cell; ESC, endothelial stem cell; hFOB, human fetal osteoblast; HSC, hepatic stem cell; iPSC, induced pluripotent stem cell; μ*g*, microgravity. Parts of the figure are drawn using pictures from Servier Medical Art (https://smart.servier.com), licensed under a Creative Commons Attribution 3.0 Unported License (https://creativecommons.org/licenses/by/3.0)

Despite this knowledge from space travelers, it is also well acknowledged that culturing of stem cells and bone cells in s‐μ*g*, using the RWV, the RCCS bioreactor, or the RPM, is a suitable technique for bone tissue engineering purposes^69^, ^70^, ^71^ and has been reviewed earlier (Table 1).^1^, ^3^ 3D cell culture environments were useful to enhance osteoblast differentiation and to engineer osseous‐like tissues from human preosteoblastic cells and human mesenchymal preosteoblastic cells (Figure 2).^69^, ^70^ The RWV bioreactor was used and patented by Clarke et al to grow mineralized 3D bone constructs.^72^ Another study demonstrated that after a 2‐week culture of fetal osteoblasts (hFOB 1.19 cell line) on an RPM, MCSs could be engineered presenting a bone‐specific morphology. The hFOB 1.19 cells are well‐characterized, stable osteoprogenitor and a well‐known model for normal osteoblast differentiation.^71^ These bone tissues may be used to study the mechanisms behind spaceflight‐related bone loss or other bone diseases such as osteonecrosis or bone injuries.^71^

A recent study demonstrated the osteogenic potential of rat bone marrow‐derived MSCs (BMSCs) in vitro.^73^ Encapsulated BMSCs differentiated into osteoblastic cells and had formed bone‐like tissue under osteogenic μ*g* bioreactor conditions.^73^

Human blood‐derived stem cells (BDSCs) were investigated recently in space on the ISS during the Italian VITA mission. Osteoblastic differentiation was induced by rapamycin.^13^ Rapamycin influenced the transcriptional activation of BDSCs toward osteogenic differentiation via elevated GATA4 and Sox17. Both factors modulate downstream transcription factors (like Runx2), critical for bone formation.^13^

Another study reported on the behavior of human BMSCs exposed to s‐μ*g*. The authors found an inhibition of proliferation and differentiation toward osteoblasts, but an increase in adipogenesis.^74^ s‐μ*g* also selected highly tumorigenic cells for survival.^74^ In addition, MSCs cultured on the SJ‐10 recoverable scientific satellite revealed an increase in the p38 MAPK activity and a de‐repression of AKT activity. The satellite flight conditions inhibited osteogenic differentiation and promoted adipogenic differentiation, even under osteogenic induction conditions.^75^

Bone MSCs exposed to μ*g* revealed alterations in regulation or functioning of the actin cytoskeleton which may cause the inhibited osteogenesis.^76^ The depolymerization of actin inhibited the osteogenic differentiation of the bone MSCs through impeding nuclear aggregation of the transcriptional coactivator with PDZ‐binding motif.^76^ Bradamante et al investigated human bone marrow stem cells, which were cultivated on the ISS for 14 days. They used vitamin D_3_ as an osteogenic differentiation inducer and compared the gene expression of in‐flight and on‐ground samples. They found that μ*g* had mainly an effect on the composition of the extracellular matrix by reducing collagens among other, while apoptosis was absent.^77^

### 4.3 Stem cells for vasculature and heart tissue engineering

#### 4.3.1 *Vasculature*

Culturing immature endothelial stem cells (ESCs) or progenitor cells (EPCs) under μ*g* conditions provide a valuable tool for therapeutic vasculogenesis and tissue regeneration and may contribute to the steadily and rapidly progressing discipline regenerative medicine. For example, EPC transplantation has become beneficial for ischemic diseases.^78^ However, the deficiency of functional EPCs in adults reflects the limiting factor for EPC transplantation as a neovascularization therapy. Microgravity was shown to improve the numbers of hematopoietic progenitor cells^79^ as well as the functions of stem cells.^60^ Hagiwara et al recently demonstrated that an initial cultivation of EPCs in μ*g* followed by cultivation under normal gravity remarkably enhanced expansion rates and angiogenic potential, including vascular endothelial growth factor (VEGF).^11^

Microgravity provides new possibilities for cell‐based therapy (reviewed by Imura et al^45^). In 2005, Chiu and colleagues cultured CD34‐positive human cord blood stem cells (CBSCs), which are involved in vascularization, in a RWV bioreactor together with or without Cytodex‐3 microcarrier beads and supplemented them with VEGF.^80^ After 4 days, the cells cultured without carrier beads assembled to tubular structures (TS) and expressed endothelial phenotypic markers such as CD31 and kinase insert domain receptor (KDR, former: Flk1), whereas only amorphic cell clusters were formed in the presence of the beads. This experiment proved that CBSCs can differentiate into a vascular endothelial phenotype under μ*g* conditions and are able to assemble into TS.

Furthermore, the μ*g* environment increases the multipotential differentiation capacity of BMSCs. Endothelium‐oriented differentiated BMSCs have been shown to express higher levels of von Willebrand factor (VWF) and CD31.^81^ Zhang et al described the differentiation of BMSCs into endothelial‐like cells after 72 hours of clinorotation. Cells expressed the endothelial‐specific markers KDR and VWF and were able to form a capillary network.^82^

Interestingly, μ*g* may stimulate cardiovascular progenitors differently depending on their age. Whereas adult cells showed elevated expression of endothelial markers and revealed unchanged or increased endothelial cell tube formation, neonatal cells showed a decline in tube formation and acquired characteristics of dedifferentiation after 6 to 7 days of clinorotation.^83^ Aboard the ISS, cytoskeletal organization, migration, and expression of DNA repair genes were affected both in neonatal and adult cardiovascular progenitors. However, only neonatal cells showed enhanced developmental markers and proliferative potential.^84^ RNA‐sequencing revealed that 2635 genes were differentially expressed among μ*g*‐exposed and control samples, including genes involved in mitochondrial metabolism.^12^ In addition, adult cardiovascular progenitors cultured under different μ*g* conditions upregulated downstream genes involved in the Hippo pathway, such as *YAP1* and *SOD2*, which may have potential benefit for cardiovascular repair.^85^

#### 4.3.2 *Heart tissue*

Biofabrication of heart tissue is a hot topic in tissue engineering. The usefulness of tissue engineered cardiac tissue is dependent among others on the differentiation status of cardiomyocytes (CMs) in vitro, on the contractility, and electrophysiological characteristics.^86^ As the growth of CMs is in part regulated by mechanical loading, the idea suggests itself that μ*g* can lead to growth changes as well. A recent study on the ISS investigated human‐induced pluripotent stem cell‐derived CMs.^10^ The authors demonstrated that long‐term cell culture (5.5 weeks) of advanced, highly specialized cell types such as human CMs is possible aboard the ISS.

Microgravity has proven to alter the CM behavior and thus can help to promote myocardial differentiation of induced pluripotent stem cells.^87^, ^88^ A combination of 3D culture and s‐μ*g* created by an RPM can be used to efficiently generate highly enriched CMs.^89^ Dissociated cardiac muscle cells cultured on 3D scaffolds in RCCS formed engineered constructs with structural and functional features resembling those of native cardiac tissue.^90^ Another study reported that simulated μ*g* provided by a 2D CN modulated the differentiation processes of embryonic stem cells.^46^ This ability can be a driving element for biofabrication methods.

In view of future space exploration plans, it is of high interest to test the effects of simulated Mars and Moon gravity using a special RPM manufactured by ADS, Leiden, and NL on the tissue engineering of heart tissue.^91^

### 4.4 Stem cells for skin repair

Different resident skin stem cell pools contribute to the maintenance and repair of the various epidermal tissues of the skin. Their development to differentiated skin cells is regulated by various factors.^92^ There are a few publications, which suggest that μ*g* may have an influence on this process. Exposing murine embryonic stem cells to parabolic flights changed 14 genes, which are involved in skin development.^93^

Human epidermal stem cells (hEpSCs) isolated from children's foreskin were successfully propagated in vitro, while hEpSCs marker and proliferative capacity are maintained. Subsequently, they were seeded in a rotary bioreactor together with microcarrier beads for 1 day, which allowed the cells to attach to the surface of the beads. Then, the sample was divided into two groups, including a rotation culture group in an RCCS and a static culture group on six‐well cell culture plates. The cells in a rotary bioreactor were prone to accumulate on the microcarrier beads forming 3D aggregates, whereas the cells in six‐well cell culture plates appeared as monolayer sheet structure on the surface of microcarrier beads. In the rotation culture group, there were a large number of cells aggregated over microcarriers beads forming 3D tissue‐like epidermis structure.^94^ Fibroblasts, an important type of skin cells, could be derived from adipose‐derived stem cells (ADSCs) exposed to μ*g*. They were isolated from fat specimens of patients. Afterward, they were cultured on a 2D CN and under 1*g* conditions. Comparing the cells after 7 days of incubation showed that the connective tissue growth factor‐induced fibroblastic differentiation of ADSCs can be modulated by exposure to s‐μ*g*. Most notably, the expression of CD44 was reduced while the production of collagen III was enhanced.^95^

### 4.5 Use of stem cells for the engineering of liver tissue

The liver is the largest inner organ and exhibits numerous unique biochemical functions. More than 500 physiological functions of the liver are reported.^96^, ^97^ The liver is involved in metabolic processes, in biochemical synthesis as well as in detoxification processes. Although it shows a high capacity of regeneration, diseases of the liver such as cirrhosis, hepatitis, and hepatocellular carcinomas account for about 2 million deaths per year worldwide.^98^ Exposure of the liver to toxicants such as alcohol due to alcohol misuse or infection with hepatitis C and B viruses and other nonalcoholic liver diseases^99^ may lead to liver cirrhosis, which is responsible for about 1.03 million deaths per year.^100^ Up to now, orthologous liver transplantation is the only treatment option available for patients suffering from such severe liver diseases. However, there is a drastic shortage of donor organs,^98^, ^101^, ^102^ so alternative methods for treating end‐stage liver disease and liver regeneration should be explored.

Majumder et al showed that only 2 hours of incubation of bipotential murine oval liver stem cell under s‐μ*g* on a 3D CN were sufficient to enhance their proliferation 2‐fold and to induce differentiation into hepatocytes within 2 to 3 days, a process probably driven by an interplay of μ*g* with BMP4/Notch1 signaling.^103^ Similarly, Zhang et al were able to generate large quantities of functional hepatocytes from mouse embryonic stems cells by culturing them in an RCCS bioreactor under s‐μ*g* in the presence of 20 ng/mL hepatocyte growth factor, 10 ng/mL recombinant human fibroblast growth factor‐4, 10 μg/mL insulin, 5 μg/mL transferrin, and 5 ng/mL selenium.^104^ Interestingly, in human hepatic and biliary tree stem/progenitor cells s‐μ*g* was shown to promote the generation of 3D spheroids and to stimulate the glycolytic metabolism, but also to favor maintenance of stemness and not the differentiation toward hepatocytes.^105^ Further steps toward reconstructing functional hepatic tissue were taken by Wang et al. Embryonic stem cells were seeded onto a biodegradable scaffold and incubated under s‐μ*g* in a rotating bioreactor.^106^ They differentiated and matured into hepatic‐like cells both in vitro as well as after a transplantation of these constructs into mice, where they remained viable and functional. Using E15.5 fetal liver cells, containing more hepatic stem/progenitor cells compared to neonatal liver cells, Ishikawa et al obtained functional hepatic tissue including mature hepatocyte and blood vessel‐like structures accompanied with bile duct‐like structures from s‐μ*g* culture conditions in an RWV, something they could not achieve using conventional 3D culture techniques.^107^ Lastly, spheroids derived from ADSCs cultivated under s‐μ*g* showed increased proliferative ability, improved multipotency differentiation capacities, and most importantly, more effective potentials to rescue liver failure in a mouse model with carbon tetrachloride‐induced acute liver failure than ADSCs derived from constant monolayer culture.^108^ Interestingly, these effects seem to be confined to s‐μ*g*, as experiments with PICM‐19 pig liver stem cells cultured for 16 days under r‐μ*g* during the STS‐126 mission revealed only minor differences between flight and ground control samples.^109^

### 4.6 General research on embryonic, pluripotent and MSCs

In general, studies on embryonic stem cells exposed to μ*g* suggested that μ*g* inhibits the stem cells' differentiation and retains their self‐renewal markers.^110^ Its inhibitory effect could be due to downregulation of a number of genes in embryonic stem cells.^46^ Similar observations were made by Kawahara et al, who were able to maintain mouse ESCs (mESCs) and embryonic bodies in an undifferentiated state over 7 days by using a 3D CN and leukemia inhibitory factor‐free culture media.^111^ A 5‐day experiment on board the SJ‐10 satellite revealed that spaceflight in general had a globally suppressive effect on gene expression in mESCs. The most affected biological processes were involved in the development of the cardiovascular system and in neurodevelopment.^112^ During the recent 15‐day TZ‐1 space mission, it was shown that r‐μ*g* prevented terminal differentiation of mESCs and promoted cell survival.^113^ Studies on murine‐induced pluripotent stem cells on the same mission seemed to suggest that r‐μ*g* promoted the expression of the pluripotency marker Oct4, therefore retaining stemness and resulting in a more dynamic behavior compared with ground controls.^114^

When cultured under s‐μ*g* in rotary bioreactors in a differentiation medium, mESC‐derived embryonic bodies showed a distinct differentiation pattern, which diverged from that observed in mESCs cultivated in spinner flasks using the same medium. While s‐μ*g* yielded more sca‐1^+^ progenitors, spinner flasks generated more c‐Kit^+^ progenitors.^115^ Using mESCs, Lei et al have shown that RCCS cultivation allows for a more controlled production of embryonic bodies with more uniform sizes and more regular shapes than those obtained from static culture techniques. They also reported that s‐μ*g* enhanced mesendoderm differentiation, whereas neuroectodermal differentiation was reduced; an effect the authors suggest was mediated by the Wnt/β‐catenin pathway.^116^, ^117^

Human adipose tissue‐derived MSCs (ADMSCs) cultured in s‐μ*g* in standard laboratory incubators alter their proliferation and differentiation. The ADMSCs were exposed for 14 days to an RPM and cultures under two oxygen concentrations: 5% and 20%. The authors showed that gene expression in RPM‐exposed cells is differently modulated depending on the oxygen concentration. They also demonstrated that simulated μ*g* influences the cytoskeleton, whereas oxygen is a key player, influencing the degree of these alterations.^118^

## 5 CONCLUSION AND FUTURE PERSPECTIVES

Exposing stem cells to μ*g* conditions increased the current knowledge about tissue engineering of various tissues such as cartilage, bone, vasculature, heart, and liver tissue (Table 1, Figure 2). In addition, stem cells have been investigated in space or with μ*g* simulators with the purpose to investigate differentiation changes and to study the relevant pathways involved in biological processes which are important for the health problems of space travelers as well as countermeasures for long‐term space exploration to Moon and Mars. Tissue engineering in μ*g* is a new technique to produce organoids, spheroids, or tissues with and without scaffolds and is useful for translational regenerative medicine (Figure 2).

Cancer research in space is currently a novel research field. Cancer research on the ISS comprises studies investigating clinical‐grade stem cells for therapeutic use or crystallizing proteins for improved drug discovery and delivery. Other cancer‐related projects study 3D cell culturing methods in space and biofabrication of tissues. This novel research should help us to rethink cancer research on Earth with the aim of developing new drugs and cancer treatment strategies.

Only a few studies investigating the impact of simulated μ*g* on CSCs were published. The results in this field are dependent on cell type and the choice of the μ*g* simulator. Non‐small lung CSCs are rescued from their quiescent state and lose their stemness when subjected to μ*g* (RPM),^15^ whereas human colorectal cancer cell HCT116 exposed to the RCCS showed an elevated but staggered autophagic flux and increased stemness, including CD133/CD44 dual positive cells.^48^ CSC generation from different cancer cell types exposed to μ*g* was successful and μ*g* simulation will be an excellent method to enrich the CD133‐positive CSCs from various cancer cell lines.^55^ This relatively small subset of cancer cells exhibits a strong capability of self‐renewal, is essentially resistant to chemotherapy, highly metastatic, and believed to be a key factor in tumor survival and progression.^119^ Therefore, tests to evaluate the efficacy of chemotherapeutic drugs on cancer cells and CSCs in vitro prior to their use in the clinic may be a novel therapy option for cancer patients. The identification of new target structures involved in CSC proliferation and differentiation may furthermore lead to the development of novel tumor‐suppressing drugs. Because of differing results obtained when using RPM, RCCS, or HFB devices, more research on different cancer types is necessary to study the biology of cancer stemness.

When comparing results from r‐ and s‐μ*g*, it should not be forgotten that there are some fundamental differences between the two conditions. Cell culture in r‐μ*g* in space takes place in an essentially force‐free environment without any perturbations of the culture medium except for the naturally occurring diffusion of nutrients and cellular waste products due to local concentration gradients. Cells on an s‐μ*g* device such as an RPM, RWV, RCSS, or CN on the other hand experience residual acceleration depending on their distance to the center of rotation, shear forces, and a constant mixture of the cell culture medium.^18^ Although these effects are not necessarily detrimental, they introduce additional factors and variations over the course of the cell culture procedure, which will eventually lead to deviations between results from r‐ and s‐μ*g* experiments. The 14‐day ESA‐SPHEROIDS space mission investigated endothelial cells in space and revealed a scaffold‐free formation of spheroids and TS in space.^8^, ^120^ Spheroid and TS formation can also be found when endothelial cells were exposed to an RPM. A similar finding was observed during the Shenzhou‐8/Simbox space mission (follicular thyroid cancer cells—FTC‐133), where spheroid formation was visible postflight. These spheroids were larger as those engineered on the RPM.^121^ The postflight analyses of the spaceflight and the RPM samples demonstrated a large number of genes similarly regulated under RPM and spaceflight conditions.^122^ Finally, μ*g* research in space and on Earth is a new technology to support the development of patient‐specific therapies and to bring new ideas to the fields of cancer research and regenerative medicine.

## CONFLICT OF INTEREST

The authors declared no potential conflicts of interest.

## AUTHOR CONTRIBUTIONS

D.G.: conception and design, manuscript writing, final approval of manuscript; M.W., T.J.C., P.R., B.P., M.L., J.B.: manuscript writing; M.E., S.K.: administrative support; M.K.: manuscript writing, graphical design.

## Data Availability

Data sharing is not applicable to this article as no new data were created or analyzed in this study.

## References

[1] Grimm D , Egli M , Krüger M , et al. Tissue engineering under microgravity conditions‐use of stem cells and specialized cells. Stem Cells Dev. 2018;27(12):787‐804. 29596037 10.1089/scd.2017.0242

[2] Strauch SM , Grimm D , Corydon TJ , et al. Current knowledge about the impact of microgravity on the proteome [in Eng]. Expert Rev Proteomic. 2019;16(1):5‐16. 10.1080/14789450.2019.1550362 30451542

[3] Ulbrich C , Wehland M , Pietsch J , et al. The impact of simulated and real microgravity on bone cells and mesenchymal stem cells. Biomed Res Int. 2014;2014:928507. 25110709 10.1155/2014/928507 PMC4119729

[4] Lewis ML , Reynolds JL , Cubano LA , Hatton JP , Lawless BD , Piepmeier EH . Spaceflight alters microtubules and increases apoptosis in human lymphocytes (Jurkat). FASEB J. 1998;12(11):1007‐1018. 9707173 10.1096/fasebj.12.11.1007

[5] Thiel CS , Tauber S , Seebacher C , et al. Real‐time 3D high‐resolution microscopy of human cells on the International Space Station. Int J Mol Sci. 2019;20(8):2033. 10.3390/ijms20082033 PMC6514950 31027161

[6] Nassef MZ , Kopp S , Wehland M , et al. Real microgravity influences the cytoskeleton and focal adhesions in human breast cancer cells. Int J Mol Sci. 2019;20:E3156. 31261642 10.3390/ijms20133156 PMC6651518

[7] Corydon TJ , Kopp S , Wehland M , et al. Alterations of the cytoskeleton in human cells in space proved by life‐cell imaging. Sci Rep. 2016;6:20043. 26818711 10.1038/srep20043 PMC4730242

[8] Krüger M , Pietsch J , Bauer J , et al. Growth of endothelial cells in space and in simulated microgravity – a comparison on the secretory level. Cell Physiol Biochem. 2019;52(5):1039‐1060. 30977987 10.33594/000000071

[9] Wang P , Tian H , Zhang J , et al. Spaceflight/microgravity inhibits the proliferation of hematopoietic stem cells by decreasing kit‐Ras/cAMP‐CREB pathway networks as evidenced by RNA‐Seq assays. FASEB J. 2019;33(5):5903‐5913. 30721627 10.1096/fj.201802413R PMC6463920

[10] Ma X , Pietsch J , Wehland M , et al. Differential gene expression profile and altered cytokine secretion of thyroid cancer cells in space. FASEB J. 2014;28(2):813‐835. 24196587 10.1096/fj.13-243287

[11] Hagiwara H , Higashibata A , Ogawa S , Kanazawa S , Mizuno H , Tanaka R . Effectiveness of endothelial progenitor cell culture under microgravity for improved angiogenic potential. Sci Rep. 2018;8(1):14239. 30250055 10.1038/s41598-018-32073-2 PMC6155294

[12] Wnorowski A , Sharma A , Chen H , et al. Effects of spaceflight on human induced pluripotent stem cell‐derived cardiomyocyte structure and function. Stem Cell Rep. 2019;13(6):960‐969. 10.1016/j.stemcr.2019.10.006 PMC6915842 31708475

[13] Gambacurta A , Merlini G , Ruggiero C , et al. Human osteogenic differentiation in space: proteomic and epigenetic clues to better understand osteoporosis. Sci Rep. 2019;9(1):8343. 31171801 10.1038/s41598-019-44593-6 PMC6554341

[14] Arun RP , Sivanesan D , Vidyasekar P , Verma RS . PTEN/FOXO3/AKT pathway regulates cell death and mediates morphogenetic differentiation of colorectal cancer cells under simulated microgravity. Sci Rep. 2017;7(1):5952. 28729699 10.1038/s41598-017-06416-4 PMC5519599

[15] Pisanu ME , Noto A , De Vitis C , et al. Lung cancer stem cell lose their stemness default state after exposure to microgravity. Biomed Res Int. 2014;2014:470253. 25276790 10.1155/2014/470253 PMC4170742

[16] Herranz R , Anken R , Boonstra J , et al. Ground‐based facilities for simulation of microgravity: organism‐specific recommendations for their use, and recommended terminology. Astrobiology. 2013;13(1):1‐17. 23252378 10.1089/ast.2012.0876 PMC3549630

[17] Eiermann P , Kopp S , Hauslage J , et al. Adaptation of a 2‐D clinostat for simulated microgravity experiments with adherent cells. Microgravity Sci Technol. 2013;25(3):153‐159.

[18] Wuest SL , Richard S , Kopp S , et al. Simulated microgravity: critical review on the use of random positioning machines for mammalian cell culture. Biomed Res Int. 2015;2015:971474. 25649075 10.1155/2015/971474 PMC4310317

[19] Borst AG , van Loon JJWA . Technology and developments for the random positioning machine, RPM. Microgravity Sci Technol. 2008;21(4):287.

[20] Benavides Damm T , Walther I , Wuest SL , et al. Cell cultivation under different gravitational loads using a novel random positioning incubator. Biotechnol Bioeng. 2014;111(6):1180‐1190. 24375199 10.1002/bit.25179 PMC4223831

[21] Jordan CT , Guzman ML , Noble M . Cancer stem cells. N Engl J Med. 2006;355(12):1253‐1261. 16990388 10.1056/NEJMra061808

[22] Lapidot T , Sirard C , Vormoor J , et al. A cell initiating human acute myeloid leukaemia after transplantation into SCID mice. Nature. 1994;367(6464):645‐648. 7509044 10.1038/367645a0

[23] Desai A , Yan Y , Gerson SL . Concise reviews: cancer stem cell targeted therapies: toward clinical success. Stem Cells Translational Medicine. 2019;8(1):75‐81. 30328686 10.1002/sctm.18-0123 PMC6312440

[24] Oktem G , Bilir A , Uslu R , et al. Expression profiling of stem cell signaling alters with spheroid formation in CD133(high)/CD44(high) prostate cancer stem cells. Oncol Lett. 2014;7(6):2103‐2109. 24932297 10.3892/ol.2014.1992 PMC4049671

[25] Atashzar MR , Baharlou R , Karami J , et al. Cancer stem cells: a review from origin to therapeutic implications. J Cell Physiol. 2020;235(2):790‐803. 31286518 10.1002/jcp.29044

[26] Vander Linden C , Corbet C . Therapeutic targeting of cancer stem cells: integrating and exploiting the acidic niche. Front Oncol. 2019;9:159. 30941310 10.3389/fonc.2019.00159 PMC6433943

[27] Afify SM , Seno M . Conversion of stem cells to cancer stem cells: undercurrent of cancer initiation. Cancers. 2019;11(3):345. 10.3390/cancers11030345 PMC6468812 30862050

[28] Ayob AZ , Ramasamy TS . Cancer stem cells as key drivers of tumour progression. J Biomed Sci. 2018;25(1):20. 29506506 10.1186/s12929-018-0426-4 PMC5838954

[29] Kusoglu A , Biray AC . Cancer stem cells: a brief review of the current status. Gene. 2019;681:80‐85. 30268439 10.1016/j.gene.2018.09.052

[30] Lorico A , Rappa G . Phenotypic heterogeneity of breast cancer stem cells. J Oncol. 2011;2011:135039. 21317983 10.1155/2011/135039 PMC3026971

[31] Al‐Hajj M , Wicha MS , Benito‐Hernandez A , et al. Prospective identification of tumorigenic breast cancer cells. Proc Natl Acad Sci USA. 2003;100(7):3983‐3988. 12629218 10.1073/pnas.0530291100 PMC153034

[32] Ginestier C , Hur MH , Charafe‐Jauffret E , et al. ALDH1 is a marker of normal and malignant human mammary stem cells and a predictor of poor clinical outcome. Cell Stem Cell. 2007;1(5):555‐567. 18371393 10.1016/j.stem.2007.08.014 PMC2423808

[33] Kopp S , Warnke E , Wehland M , et al. Mechanisms of three‐dimensional growth of thyroid cells during long‐term simulated microgravity. Sci Rep. 2015;5:16691. 26576504 10.1038/srep16691 PMC4649336

[34] Kopp S , Slumstrup L , Corydon TJ , et al. Identifications of novel mechanisms in breast cancer cells involving duct‐like multicellular spheroid formation after exposure to the random positioning machine. Sci Rep. 2016;6:26887. 27230828 10.1038/srep26887 PMC4882535

[35] Lelkes PI , Galvan DL , Hayman GT , et al. Simulated microgravity conditions enhance differentiation of cultured PC12 cells towards the neuroendocrine phenotype. In Vitro Cell Dev Biol Anim. 1998;34(4):316‐325. 9590505 10.1007/s11626-998-0008-y

[36] Hybel TE , Dietrichs D , Sahana J , et al. Simulated microgravity influences VEGF, MAPK, and PAM signaling in prostate cancer cells. Int J Mol Sci. 2020;21(4):1263. 10.3390/ijms21041263 PMC7072928 32070055

[37] Masiello MG , Cucina A , Proietti S , et al. Phenotypic switch induced by simulated microgravity on MDA‐MB‐231 breast cancer cells. Biomed Res Int. 2014;2014:652434. 25215287 10.1155/2014/652434 PMC4151603

[38] Cirello V , Vaira V , Grassi ES , et al. Multicellular spheroids from normal and neoplastic thyroid tissues as a suitable model to test the effects of multikinase inhibitors. Oncotarget. 2017;8(6):9752‐9766. 28039458 10.18632/oncotarget.14187 PMC5354768

[39] Shi Y , Fu X , Hua Y , Han Y , Lu Y , Wang J . The side population in human lung cancer cell line NCI‐H460 is enriched in stem‐like cancer cells. PLoS One. 2012;7(3):e33358. 22428030 10.1371/journal.pone.0033358 PMC3302833

[40] Sahebi R , Aghaei M , Halvaei S , Alizadeh A . The role of microgravity in cancer: a dual‐edge sword. Multidiscip Cancer Investig. 2017;1(3):1‐5.

[41] Chung JH , Ahn CB , Son KH , et al. Simulated microgravity effects on nonsmall cell lung cancer cell proliferation and migration. Aerosp Med Hum Perform. 2017;88(2):82‐89. 28095951 10.3357/AMHP.4647.2017

[42] Ahn CB , Lee JH , Han DG , et al. Simulated microgravity with floating environment promotes migration of non‐small cell lung cancers. Sci Rep. 2019;9(1):14553. 31601869 10.1038/s41598-019-50736-6 PMC6787256

[43] Chin AM , Hill DR , Aurora M , Spence JR . Morphogenesis and maturation of the embryonic and postnatal intestine. Semin Cell Dev Biol. 2017;66:81‐93. 28161556 10.1016/j.semcdb.2017.01.011 PMC5487846

[44] Hata M , Hayakawa Y , Koike K . Gastric stem cell and cellular origin of cancer. Biomedicines. 2018;6(4):100. 10.3390/biomedicines6040100 PMC6315982 30384487

[45] Imura T , Otsuka T , Kawahara Y , Yuge L . "Microgravity" as a unique and useful stem cell culture environment for cell‐based therapy. Regen Ther. 2019;12:2‐5. 31890760 10.1016/j.reth.2019.03.001 PMC6933149

[46] Shinde V , Brungs S , Henry M , et al. Simulated microgravity modulates differentiation processes of embryonic stem cells. Cell Physiol Biochem. 2016;38(4):1483‐1499. 27035921 10.1159/000443090

[47] Allen DL , Bandstra ER , Harrison BC , et al. Effects of spaceflight on murine skeletal muscle gene expression. J Appl Physiol (1985). 2009;106(2):582‐595. 19074574 10.1152/japplphysiol.90780.2008 PMC2644242

[48] Arun RP , Sivanesan D , Patra B , Varadaraj S , Verma RS . Simulated microgravity increases polyploid giant cancer cells and nuclear localization of YAP. Sci Rep. 2019;9(1):10684. 31337825 10.1038/s41598-019-47116-5 PMC6650394

[49] Devarasetty M , Wang E , Soker S , Skardal A . Mesenchymal stem cells support growth and organization of host‐liver colorectal‐tumor organoids and possibly resistance to chemotherapy. Biofabrication. 2017;9(2):021002. 28589925 10.1088/1758-5090/aa7484 PMC7945991

[50] Zuba‐Surma EK , Kucia M , Wu W , et al. Very small embryonic‐like stem cells are present in adult murine organs: ImageStream‐based morphological analysis and distribution studies. Cytometry A. 2008;73A(12):1116‐1127. 18951465 10.1002/cyto.a.20667 PMC2646009

[51] Starzynska T , Dabkowski K , Blogowski W , et al. An intensified systemic trafficking of bone marrow‐derived stem/progenitor cells in patients with pancreatic cancer. J Cell Mol Med. 2013;17(6):792‐799. 23672538 10.1111/jcmm.12065 PMC3823183

[52] Zuellig RA , Cavallari G , Gerber P , et al. Improved physiological properties of gravity‐enforced reassembled rat and human pancreatic pseudo‐islets. J Tissue Eng Regen Med. 2017;11(1):109‐120. 24737702 10.1002/term.1891

[53] Samuelson L , Gerber DA . Improved function and growth of pancreatic cells in a three‐dimensional bioreactor environment. Tissue Eng Part C Methods. 2013;19(1):39‐47. 22712746 10.1089/ten.TEC.2012.0236

[54] Qureshi KM , Lee J , Paget MB , et al. Low gravity rotational culture and the integration of immunomodulatory stem cells reduce human islet allo‐reactivity. Clin Transplant. 2015;29(1):90‐98. 25382449 10.1111/ctr.12488

[55] Kelly SE , Di Benedetto A , Greco A , et al. Rapid selection and proliferation of CD133+ cells from cancer cell lines: chemotherapeutic implications. PLoS One. 2010;5(4):e10035. 20386701 10.1371/journal.pone.0010035 PMC2851647

[56] Hunziker EB . Articular cartilage repair: basic science and clinical progress. A review of the current status and prospects. Osteoarthr Cartil. 2002;10(6):432‐463. 12056848 10.1053/joca.2002.0801

[57] Ohyabu Y , Kida N , Kojima H , Taguchi T , Tanaka J , Uemura T . Cartilaginous tissue formation from bone marrow cells using rotating wall vessel (RWV) bioreactor. Biotechnol Bioeng. 2006;95(5):1003‐1008. 16986169 10.1002/bit.20892

[58] Wu X , Li SH , Lou LM , Chen ZR . The effect of the microgravity rotating culture system on the chondrogenic differentiation of bone marrow mesenchymal stem cells. Mol Biotechnol. 2013;54(2):331‐336. 22669584 10.1007/s12033-012-9568-x

[59] Luo W , Xiong W , Qiu M , Lv Y , Li Y , Li F . Differentiation of mesenchymal stem cells towards a nucleus pulposus‐like phenotype utilizing simulated microgravity *in vitro* . J Huazhong Univ Sci Technolog Med Sci. 2011;31(2):199‐203. 21505985 10.1007/s11596-011-0252-3

[60] Yuge L , Kajiume T , Tahara H , et al. Microgravity potentiates stem cell proliferation while sustaining the capability of differentiation. Stem Cells Dev. 2006;15(6):921‐929. 17253953 10.1089/scd.2006.15.921

[61] Yu B , Yu D , Cao L , et al. Simulated microgravity using a rotary cell culture system promotes chondrogenesis of human adipose‐derived mesenchymal stem cells via the p38 MAPK pathway. Biochem Biophys Res Commun. 2011;414(2):412‐418. 21971552 10.1016/j.bbrc.2011.09.103

[62] Yin H , Wang Y , Sun Z , et al. Induction of mesenchymal stem cell chondrogenic differentiation and functional cartilage microtissue formation for *in vivo* cartilage regeneration by cartilage extracellular matrix‐derived particles. Acta Biomater. 2016;33:96‐109. 26802442 10.1016/j.actbio.2016.01.024

[63] Yin H , Wang Y , Sun X , et al. Functional tissue‐engineered microtissue derived from cartilage extracellular matrix for articular cartilage regeneration. Acta Biomater. 2018;77:127‐141. 30030172 10.1016/j.actbio.2018.07.031

[64] Weiss WM , Mulet‐Sierra A , Kunze M , Jomha NM , Adesida AB . Coculture of meniscus cells and mesenchymal stem cells in simulated microgravity. NPJ Microgravity. 2017;3:28. 29147680 10.1038/s41526-017-0032-x PMC5681589

[65] Chen L , Liu G , Li W , Wu X . Chondrogenic differentiation of bone marrow‐derived mesenchymal stem cells following transfection with Indian hedgehog and sonic hedgehog using a rotary cell culture system. Cell Mol Biol Lett. 2019;24:16. 30858866 10.1186/s11658-019-0144-2 PMC6390628

[66] Mayer‐Wagner S , Hammerschmid F , Redeker JI , et al. Simulated microgravity affects chondrogenesis and hypertrophy of human mesenchymal stem cells. Int Orthop. 2014;38(12):2615‐2621. 25030964 10.1007/s00264-014-2454-3

[67] Mayer‐Wagner S , Hammerschmid F , Blum H , et al. Effects of single and combined low frequency electromagnetic fields and simulated microgravity on gene expression of human mesenchymal stem cells during chondrogenesis. Arch Med Sci. 2018;14(3):608‐616. 29765449 10.5114/aoms.2016.59894 PMC5949910

[68] Grimm D , Grosse J , Wehland M , et al. The impact of microgravity on bone in humans. Bone. 2016;87:44‐56. 27032715 10.1016/j.bone.2015.12.057

[69] Boehrs J , Zaharias RS , Laffoon J , Ko YJ , Schneider GB . Three‐dimensional culture environments enhance osteoblast differentiation. J Prosthodont. 2008;17(7):517‐521. 18573152 10.1111/j.1532-849X.2008.00330.x

[70] Schneider GB , Boehrs JK , Hoopes JV , et al. Use of 3‐dimensional environments to engineer osseous‐like tissue. J Dev Biol Tissue Eng. 2011;3(4):42‐47.

[71] Mann V , Grimm D , Corydon TJ , et al. Changes in human foetal osteoblasts exposed to the random positioning machine and bone construct tissue engineering. Int J Mol Sci. 2019;20(6):1357. 10.3390/ijms20061357 PMC6471706 30889841

[72] Clarke MS , Sundaresan A , Vanderburg CR , et al. A three‐dimensional tissue culture model of bone formation utilizing rotational co‐culture of human adult osteoblasts and osteoclasts. Acta Biomater. 2013;9(8):7908‐7916. 23664885 10.1016/j.actbio.2013.04.051

[73] Koc Demir A , Elcin AE , Elcin YM . Osteogenic differentiation of encapsulated rat mesenchymal stem cells inside a rotating microgravity bioreactor: in vitro and in vivo evaluation. Cytotechnology. 2018;70(5):1375‐1388. 29943233 10.1007/s10616-018-0230-8 PMC6214859

[74] Li L , Zhang C , Chen JL , Hong FF , Chen P , Wang JF . Effects of simulated microgravity on the expression profiles of RNA during osteogenic differentiation of human bone marrow mesenchymal stem cells. Cell Prolif. 2019;52(2):e12539. 30397970 10.1111/cpr.12539 PMC6496301

[75] Zhang C , Li L , Jiang Y , et al. Space microgravity drives transdifferentiation of human bone marrow‐derived mesenchymal stem cells from osteogenesis to adipogenesis. FASEB J. 2018;32(8):4444‐4458. 29533735 10.1096/fj.201700208RR

[76] Chen Z , Luo Q , Lin C , et al. Simulated microgravity inhibits osteogenic differentiation of mesenchymal stem cells via depolymerizing F‐actin to impede TAZ nuclear translocation. Sci Rep. 2016;6:30322. 27444891 10.1038/srep30322 PMC4957213

[77] Bradamante S , Rivero D , Barenghi L , et al. SCD – stem cell differentiation toward osteoblast onboard the International Space Station. Microgravity Sci Technol. 2018;30(5):713‐729.

[78] Chong MSK , Ng WK , Chan JKY . Concise review: endothelial progenitor cells in regenerative medicine: applications and challenges. Stem Cells Translational Medicine. 2016;5(4):530‐538. 26956207 10.5966/sctm.2015-0227 PMC4798740

[79] Kajiume T , Yuge L , Kawahara Y , et al. Floating culture promotes the maintenance of hematopoietic stem cells. FEBS Lett. 2007;581(24):4645‐4650. 17825827 10.1016/j.febslet.2007.08.057

[80] Chiu B , Wan JZM , Abley D , et al. Induction of vascular endothelial phenotype and cellular proliferation from human cord blood stem cells cultured in simulated microgravity [in English]. Acta Astronaut. 2005;56(9–12):918‐922. 15835045 10.1016/j.actaastro.2005.01.018

[81] Wang N , Wang H , Chen J , et al. The simulated microgravity enhances multipotential differentiation capacity of bone marrow mesenchymal stem cells. Cytotechnology. 2014;66(1):119‐131. 23579245 10.1007/s10616-013-9544-8 PMC3886541

[82] Zhang X , Nan Y , Wang H , et al. Model microgravity enhances endothelium differentiation of mesenchymal stem cells. Naturwissenschaften. 2013;100(2):125‐133. 23229853 10.1007/s00114-012-1002-5

[83] Fuentes TI , Appleby N , Raya M , et al. Simulated microgravity exerts an age‐dependent effect on the differentiation of cardiovascular progenitors isolated from the human heart. PLoS One. 2015;10(7):e0132378. 26161778 10.1371/journal.pone.0132378 PMC4498633

[84] Baio J , Martinez AF , Silva I , et al. Cardiovascular progenitor cells cultured aboard the International Space Station exhibit altered developmental and functional properties. NPJ Microgravity. 2018;4:13. 30062101 10.1038/s41526-018-0048-x PMC6062551

[85] Camberos V , Baio J , Bailey L , et al. Effects of spaceflight and simulated microgravity on YAP1 expression in cardiovascular progenitors: implications for cell‐based repair. Int J Mol Sci. 2019;20(11):2742. 10.3390/ijms20112742 PMC6600678 31167392

[86] Zimmermann WH , Schneiderbanger K , Schubert P , et al. Tissue engineering of a differentiated cardiac muscle construct. Circ Res. 2002;90(2):223‐230. 11834716 10.1161/hh0202.103644

[87] Islas JF , Abbasgholizadeh R , Dacso C , et al. Beta‐adrenergic stimuli and rotating suspension culture enhance conversion of human adipogenic mesenchymal stem cells into highly conductive cardiac progenitors. J Tissue Eng Regen Med. 2020;14(2):306‐318. 31821703 10.1002/term.2994

[88] Li H , Zhu H , Zhang F , et al. Spaceflight promoted myocardial differentiation of induced pluripotent stem cells: results from Tianzhou‐1 space Mission. Stem Cells Dev. 2019;28(6):357‐360. 30654722 10.1089/scd.2018.0240

[89] Jha R , Wu Q , Singh M , et al. Simulated microgravity and 3D culture enhance induction, viability, proliferation and differentiation of cardiac progenitors from human pluripotent stem cells. Sci Rep. 2016;6:30956. 27492371 10.1038/srep30956 PMC4974658

[90] Liu X , Wang CY , Guo XM , OuYang W . Experimental study of cardiac muscle tissue engineering in bioreactor. Zhongguo Yi Xue Ke Xue Yuan Xue Bao. 2003;25(1):7‐12. 12905598

[91] Manzano A , Herranz R , den Toom LA , et al. Novel, Moon and Mars, partial gravity simulation paradigms and their effects on the balance between cell growth and cell proliferation during early plant development. NPJ Microgravity. 2018;4:9. 29644337 10.1038/s41526-018-0041-4 PMC5884789

[92] Blanpain C , Fuchs E . Epidermal homeostasis: a balancing act of stem cells in the skin. Nat Rev Mol Cell Biol. 2009;10(3):207‐217. 19209183 10.1038/nrm2636 PMC2760218

[93] Acharya A , Brungs S , Henry M , et al. Modulation of differentiation processes in murine embryonic stem cells exposed to parabolic flight‐induced acute Hypergravity and microgravity. Stem Cells Dev. 2018;27(12):838‐847. 29630478 10.1089/scd.2017.0294 PMC5995265

[94] Lei XH , Ning LN , Cao YJ , et al. NASA‐approved rotary bioreactor enhances proliferation of human epidermal stem cells and supports formation of 3D epidermis‐like structure. PLoS One. 2011;6(11):e26603. 22096490 10.1371/journal.pone.0026603 PMC3212516

[95] Ebnerasuly F , Hajebrahimi Z , Tabaie SM , Darbouy M . Effect of simulated microgravity conditions on differentiation of adipose derived stem cells towards fibroblasts using connective tissue growth factor. Iran J Biotechnol. 2017;15(4):241‐251. 29845076 10.15171/ijb.1747 PMC5903911

[96] Naruse K , Tang W , Makuuch M . Artificial and bioartificial liver support: a review of perfusion treatment for hepatic failure patients. World J Gastroenterol. 2007;13(10):1516‐1521. 17461442 10.3748/wjg.v13.i10.1516 PMC4146892

[97] Bhatia SN , Underhill GH , Zaret KS , et al. Cell and tissue engineering for liver disease. Sci Trans Med. 2014;6(245):245sr242‐245sr242. 10.1126/scitranslmed.3005975 PMC4374645 25031271

[98] Asrani SK , Devarbhavi H , Eaton J , Kamath PS . Burden of liver diseases in the world. J Hepatol. 2019;70(1):151‐171. 30266282 10.1016/j.jhep.2018.09.014

[99] Zezos P , Renner EL . Liver transplantation and non‐alcoholic fatty liver disease. World J Gastroenterol. 2014;20(42):15532‐15538. 25400437 10.3748/wjg.v20.i42.15532 PMC4229518

[100] Tsochatzis EA , Bosch J , Burroughs AK . Liver cirrhosis. Lancet. 2014;383(9930):1749‐1761. 24480518 10.1016/S0140-6736(14)60121-5

[101] Husen P , Hornung J , Benko T , et al. Risk factors for high mortality on the liver transplant waiting list in times of organ shortage: a single‐center analysis. Ann Transplant. 2019;24:242‐251. 31048668 10.12659/AOT.914246 PMC6519305

[102] Czigany Z , Lurje I , Tolba RH , Neumann UP , Tacke F , Lurje G . Machine perfusion for liver transplantation in the era of marginal organs—new kids on the block. Liver Int. 2019;39(2):228‐249. 30129192 10.1111/liv.13946

[103] Majumder S , Siamwala JH , Srinivasan S , et al. Simulated microgravity promoted differentiation of bipotential murine oval liver stem cells by modulating BMP4/Notch1 signaling. J Cell Biochem. 2011;112(7):1898‐1908. 21433062 10.1002/jcb.23110

[104] Zhang S , Zhang Y , Chen L , et al. Efficient large‐scale generation of functional hepatocytes from mouse embryonic stem cells grown in a rotating bioreactor with exogenous growth factors and hormones. Stem Cell Res Ther. 2013;4(6):145. 24294908 10.1186/scrt356 PMC4054944

[105] Costantini D , Overi D , Casadei L , et al. Simulated microgravity promotes the formation of tridimensional cultures and stimulates pluripotency and a glycolytic metabolism in human hepatic and biliary tree stem/progenitor cells. Sci Rep. 2019;9(1):5559. 30944365 10.1038/s41598-019-41908-5 PMC6447605

[106] Wang Y , Zhang Y , Zhang S , et al. Rotating microgravity‐bioreactor cultivation enhances the hepatic differentiation of mouse embryonic stem cells on biodegradable polymer scaffolds. Tissue Eng Part A. 2012;18(21–22):2376‐2385. 22712633 10.1089/ten.TEA.2012.0097

[107] Ishikawa M , Sekine K , Okamura A , et al. Reconstitution of hepatic tissue architectures from fetal liver cells obtained from a three‐dimensional culture with a rotating wall vessel bioreactor. J Biosci Bioeng. 2011;111(6):711‐718. 21402492 10.1016/j.jbiosc.2011.01.019

[108] Zhang S , Liu P , Chen L , Wang Y , Wang Z , Zhang B . The effects of spheroid formation of adipose‐derived stem cells in a microgravity bioreactor on stemness properties and therapeutic potential. Biomaterials. 2015;41:15‐25. 25522961 10.1016/j.biomaterials.2014.11.019

[109] Talbot NC , Caperna TJ , Blomberg L , Graninger PG , Stodieck LS . The effects of space flight and microgravity on the growth and differentiation of PICM‐19 pig liver stem cells. In Vitro Cell Dev Biol Anim. 2010;46(6):502‐515. 20333478 10.1007/s11626-010-9302-6

[110] Blaber EA , Finkelstein H , Dvorochkin N , et al. Microgravity reduces the differentiation and regenerative potential of embryonic stem cells. Stem Cells Dev. 2015;24(22):2605‐2621. 26414276 10.1089/scd.2015.0218 PMC4652210

[111] Kawahara Y , Manabe T , Matsumoto M , Kajiume T , Matsumoto M , Yuge L . LIF‐free embryonic stem cell culture in simulated microgravity. PLoS One. 2009;4(7):e6343. 19626124 10.1371/journal.pone.0006343 PMC2710515

[112] An L , Li Y , Fan Y , et al. The trends in global gene expression in mouse embryonic stem cells during spaceflight. Front Genet. 2019;10:768. 31552089 10.3389/fgene.2019.00768 PMC6743352

[113] Lei X , Cao Y , Zhang Y , et al. Effect of microgravity on proliferation and differentiation of embryonic stem cells in an automated culturing system during the TZ‐1 space mission. Cell Prolif. 2018;51(5):e12466. 29999554 10.1111/cpr.12466 PMC6528932

[114] Zhou J , Dong XH , Zhang FZ , et al. Real microgravity condition promoted regeneration capacity of induced pluripotent stem cells during the TZ‐1 space mission. Cell Prolif. 2019;52(3):e12574. 30724402 10.1111/cpr.12574 PMC6536455

[115] Fridley KM , Fernandez I , Li MT , et al. Unique differentiation profile of mouse embryonic stem cells in rotary and stirred tank bioreactors. Tissue Eng Part A. 2010;16(11):3285‐3298. 20528675 10.1089/ten.tea.2010.0166 PMC2965195

[116] Lei X , Deng Z , Zhang H , et al. Rotary suspension culture enhances mesendoderm differentiation of embryonic stem cells through modulation of Wnt/beta‐catenin pathway. Stem Cell Rev Rep. 2014;10(4):526‐538. 24793926 10.1007/s12015-014-9511-6

[117] Lei X , Deng Z , Duan E . Uniform embryoid body production and enhanced mesendoderm differentiation with murine embryonic stem cells in a rotary suspension bioreactor. Methods Mol Biol. 2016;1502:63‐75. 27115505 10.1007/7651_2016_354

[118] Versari S , Klein‐Nulend J , van Loon J , Bradamante S . Influence of oxygen in the cultivation of human mesenchymal stem cells in simulated microgravity: an explorative study. Microgravity Sci Technol. 2013;25(1):59‐66.

[119] Liou GY . CD133 as a regulator of cancer metastasis through the cancer stem cells. Int J Biochem Cell Biol. 2019;106:1‐7. 30399449 10.1016/j.biocel.2018.10.013 PMC6309463

[120] Pietsch J , Gass S , Nebuloni S , et al. Three‐dimensional growth of human endothelial cells in an automated cell culture experiment container during the SpaceX CRS‐8 ISS space mission ‐ the SPHEROIDS project. Biomaterials. 2017;124:126‐156. 28199884 10.1016/j.biomaterials.2017.02.005

[121] Pietsch J , Ma X , Wehland M , et al. Spheroid formation of human thyroid cancer cells in an automated culturing system during the Shenzhou‐8 space mission. Biomaterials. 2013;34(31):7694‐7705. 23866977 10.1016/j.biomaterials.2013.06.054

[122] Ma X , Pietsch J , Wehland M , et al. Differential gene expression profile and altered cytokine secretion of thyroid cancer cells in space. FASEB J. 2014;28(2):813‐835. 24196587 10.1096/fj.13-243287

